# Involvement of Epigenetic Promoter DNA Methylation of miR-124 in the Pathogenesis of HIV-1-Associated Neurocognitive Disorders

**DOI:** 10.1177/2516865718806904

**Published:** 2018-10-14

**Authors:** Shilpa Buch, Palsamy Periyasamy, Minglei Guo

**Affiliations:** Department of Pharmacology and Experimental Neuroscience, University of Nebraska Medical Center, Omaha, NE, USA

**Keywords:** DNA methylation, MECP2, epigenetics, miR-124, microglia, neuroinflammation

## Abstract

Despite the efficacy of combination antiretroviral therapy (cART) in controlling viremia, the central nervous system (CNS) continues to harbor viral reservoirs. The persistence of low-level virus replication leads to the accumulation of early viral proteins, including HIV-1 Transactivator of transcription (HIV-1 Tat) protein. Based on the premise that cART does not impact levels of HIV-1 Tat, and since the CNS is inaccessible to the cART regimens, HIV-1-Tat-mediated neuroinflammation has been implicated as an underlying mediator of HIV-1-associated neurocognitive disorders (HAND). The mechanism(s) underlying the pathogenesis of HAND, however, remain less understood. Understanding the epigenetic/molecular mechanism(s) by which viral proteins such as HIV-1 Tat activate microglia is thus of paramount importance. The study published by Periyasamy et al provides new mechanistic insights into the role of HIV-1-Tat-mediated DNA methylation of miR-124 promoter in regulating microglial activation via the MECP2-STAT3 signaling axis. Furthermore, the authors have also reported that exposure of mouse primary microglial cells to HIV-1 Tat notably increased DNA methylation of primary miR-124-1 and primary miR-124-2 promoters (with no change in primary miR-124-3), resulting in turn to downregulated expression of both primary miR-124-1 and primary miR-124-2 as well as mature miR-124 in mouse primary microglial cells. The authors also examined the involvement of MECP2-STAT3 signaling in HIV-1-Tat-mediated microglial activation. Based on these novel findings, it is evident that dysregulation of miR-124 is involved in the pathogenesis of HAND and that restoration of miR-124 could serve as an adjunctive treatment for dampening neuroinflammation associated with HAND.

**Comment on:** Periyasamy P, Thangaraj A, Guo ML, Hu G, Callen S, Buch S. Epigenetic promoter DNA methylation of miR-124 promotes HIV-1 Tat-mediated microglial activation via MECP2-STAT3 axis. J Neurosci. 2018;38:5367-5383. doi:10.1523/JNEUROSCI.3474-17.2018. PubMed PMID:29760177; PubMed Central PMCID:PMC5990983. https://www.ncbi.nlm.nih.gov/pubmed/29760177

In the era of combination antiretroviral therapy (cART), HIV-1 infection has transitioned from a fatal disease into a chronic and a more manageable disorder. Paradoxically, however, the prevalence of HIV-1-associated neurological disorders (HAND) has been on the rise owing to the increased life span of infected individuals. Almost 30% to 60% of the HIV-1-infected individuals on cART are diagnosed with HAND with symptoms ranging from asymptomatic to minor cognitive motor disorders.^1^ The mechanism(s) contributing to HAND implicate the role of underlying neuroinflammation involving microglial activation.^2^ Intriguingly, in the presence of cART, while there is dramatic suppression of HIV replication in the periphery, the persistence of HIV-1 proteins such as Transactivator of Transcription (HIV-1 Tat) protein continues in tissues such as the brain. Although numerous studies point to the role of HIV-1 Tat in activating microglia both in vitro as well as in vivo,^3^ detailed molecular and epigenetic mechanism(s) underlying this process remain unexplored.

Epigenetic regulation, one of the most-conserved mechanisms, plays a crucial role in gene expression and cellular activities. Included in this regulatory process is gene regulation by small non-coding RNAs termed as microRNAs (miR) that can modulate hundreds of genes simultaneously to exert profound effects on cellular activities such as proliferation, differentiation, and apoptosis.^4^ The brain is an essential organ within which miRNAs play crucial roles in controlling gene expression and neuronal activities. Intriguingly, dysregulation of several brain-enriched miRNAs has been shown to be closely associated with microglial activation.^5,6^ Along these lines, HIV-1 Tat has also been shown to regulate the expression profiles of miRNAs.^7^ One such miR regulated by HIV-1 Tat includes miR-124, which is highly expressed in the brain (microglia and neurons) and is critical for maintaining microglial quiescence and homeostasis of neuronal plasticity in the adult brain.^8,9^

To explore the possible role of miR-124 in microglial activation, the authors first performed miR microarray to determine the expression profile of various dysregulated miRs in the basal ganglia of saline and simian immunodeficiency virus (SIV)-infected rhesus macaques. As mentioned earlier, miR-124 is a crucial miR responsible for microglia quiescence; its downregulation leads to increased microglial activation.^9^ In this study, Periyasamy et al^10^ primarily focused on the involvement of miR-124 in microglial activation both during SIV infection and following exposure of microglial to HIV-1 Tat (as a surrogate of HIV infection). SIV infection of rhesus macaques resulted in downregulation of miR-124 in the basal ganglia, thereby indicating a negative relationship between miR-124 and microglial activation.

This study further demonstrated increased 5-mC levels (an indicator of global DNA methylation) as well as increased expression of DNA methylation enzymes such as DNMT1, DNMT3a, and DNMT3b in the homogenates isolated from basal ganglia of SIV-infected rhesus macaques. Additionally, findings from this study also demonstrated that exposure of mouse primary microglial cells to HIV-1 Tat protein resulted in downregulated expression of both mature miR-124 and the primary miR-124-1 and primary miR-124-2 (but not primary miR-124-3). The authors speculated that the possible explanation for the lack of change in HIV-1-Tat-mediated DNA methylation of the primary miR-124-3 promoter could likely be attributed to its role as a compensatory check that is functional only in the absence of two other miRs. Further detailed studies are warranted to explore this speculation.

Using various pharmacological and gene silencing approaches to knockdown DNMT1, the authors ruled out any association between miR-124 expression and DNA methylation enzymes in microglia exposed to HIV-1 Tat. These findings are in agreement with other reports demonstrating that both HIV-1 infection and HIV-1 Tat can induce the expression of DNMTs in lymphomas, thereby leading to increased methylation of genomic DNA with dysregulated gene/miR expression.^11^ Accumulating evidence has also demonstrated downregulation of miR-124 both in the archival brain tissues and in the cerebrospinal fluid of subjects who are HIV-positive and have HIV-1 encephalitis.^12^

This study, for the first time, reports a novel 3′-UTR target of miR-124 such as MECP2. It is well known that MECP2 is a transcriptional repressor that selectively binds to the methylated DNA and forms complexes with several other repressor proteins, thereby silencing the expression of specific genes. It is also documented that MECP2 can suppress the nuclear miR processing by regulating the DGCR8/Drosha complex resulting in decreased expression of several mature miRs including miR-124.^13^ Intriguingly, it has also been demonstrated that phosphorylated MECP2 (Ser80) directly binds to the DGCR8 and releases Drosha from DGCR8, thereby blocking miR biogenesis.^13^ Similar to these published reports, this study also demonstrated that in microglia, HIV-1-Tat-mediated downregulation of miR-124 resulted in increased expression of its 3′-UTR target protein MECP2 as well as its phosphorylated form of MECP2 (Ser80). Furthermore, this study also demonstrated that increased expression of phosphorylated MECP2 (Ser80) could block the miR biogenesis machinery, leading ultimately to further downregulation of miR-124 through a negative regulatory feedback axis.

The authors also investigated the involvement of STAT3 signaling in HIV Tat/miR-124 axis-mediated activation of mouse primary microglial cells. The authors convincingly showed that in microglial cells, HIV-1-Tat-mediated downregulation of miR-124 upregulated the expression of both STAT3 and its activated, phosphorylated form. Lending credence to the above findings are findings by other investigators demonstrating that peripheral administration of miR-124 in an experimental murine autoimmune encephalomyelitis model resulted in the deactivation of macrophages, reduced activation of myelin-specific T cells, and marked suppression of disease progression via downregulation of STAT3 signaling.^14^ It is well established that proinflammatory cytokines secreted by activated glia or damaged cells play a fundamental role in triggering neuroinflammation. Accordingly, it is possible that HIV-1-Tat-mediated microglial activation leads to overexpression of proinflammatory cytokines, thereby further underpinning the detrimental role of HIV-1 Tat in mediating microglial activation.

Overall, the authors have demonstrated that HIV-1-Tat-mediated downregulation of miR-124 involves DNA methylation of primary miR-124-1 and primary miR-124-2 promoters. Furthermore, downregulated miR-124 regulated the MECP2-STAT3 signaling axis, leading, in turn, to increased expression of interleukin (IL)-6and ensuing microglial activation. These novel results provide functional insights into the interaction of HIV-1 Tat with miR-124 promoter DNA methylation and MECP2-STAT3-IL6 signaling axis, further implicating that inhibition of DNMTs/MECP2, as well as overexpression of miR-124, could be developed as future adjunctive approaches for dampening HIV-1-Tat-mediated microglial activation.

These findings could have broad implications in other neuroinflammatory agents, such as drugs of abuse like cocaine, which have also been shown to downregulate miR-124, both dose- and time-dependently, in BV2 microglial cells as well as rat primary microglia.^15^ In the brain, miR-124 is highly expressed not only in microglia but also in neurons, with high levels of miR-124 playing critical roles in the maintenance of homeostasis of neuronal plasticity as well. Based on the close interactions of microglia and neurons in the central nervous system (CNS), future investigations on the role of miR-124 in HIV-1 Tat-mediated neuronal injury are warranted. In our unpublished findings, we have shown that HIV-1 Tat exposure of rat primary neurons also resulted in significantly decreased expression of miR-124. In this study, HIV-1 Tat upregulated Beclin1 and LC3II puncta formation—two markers of autophagy. Interestingly, miR-124 directly regulated the expression of Beclin1, and furthermore, miR-124 overexpression reversed HIV-1-Tat-mediated upregulation of Beclin1 and autophagosome formation in primary neurons. Moreover, lentiviral-mediated overexpression of miR-124 blocked HIV-1 Tat-mediated reduction of spine density in vitro. The association among miR-124, Beclin1, and spine density was further confirmed in a rodent HAND model in vivo. These novel findings demonstrated that HIV-1 could also decrease neuronal miR-124 levels epigenetically via regulation of the autophagy process that was linked to changes in neuronal morphology (decreased spine density).

In summary, our findings implicate a critical role of epigenetic regulation of miR-124 in the pathogenesis of HAND in the context of neuroinflammation. This study sets the stage for future assessment of other miRs in the pathogenesis of HAND. Ramifications of this study could also involve evaluation of the role of anti-miRs as adjunctive treatment strategies for HAND.
